# Cumulative incidence rates for CNS and non-CNS progression in two phase II studies of alectinib in *ALK*-positive NSCLC

**DOI:** 10.1038/bjc.2017.395

**Published:** 2017-11-16

**Authors:** Shirish Gadgeel, Alice T Shaw, Fabrice Barlesi, Lucio Crinò, James Chih-Hsin Yang, Anne-Marie C Dingemans, Dong-Wan Kim, Filippo de Marinis, Mathias Schulz, Shiyao Liu, Ravindra Gupta, Ahmed Kotb, Sai-Hong Ignatius Ou

**Affiliations:** 1 Department of Internal Medicine, Division of Hematology and Oncology, The University of Michigan, 1500 E. Medical Center Drive, 7217CC, Ann Arbor, MI 48109, USA; 2 Department of Medicine, Massachusetts General Hospital, 55 Fruit Street, Boston, MA 02114, USA; 3 Multidisciplinary Oncology and Therapeutic Innovations Department, Aix-Marseille University, Assistance Publique Hôpitaux de Marseille, 58 Boulevard Charles Livon, Marseille 13284, France; 4 Department of Oncology, Istituto Scientifico Romagnolo per lo Studio e la cura dei Tumori IRCCS Meldola, Meldola Province of Forlì-Cesena 47014, Italy; 5 Department of Oncology, National Taiwan University Hospital and National Taiwan University Cancer Center, No. 7, Zhongshan South Road, Zhongzheng District, Taipei City, Taiwan; 6 Department of Pulmonology, Maastricht University Medical Center, PO Box 5800, Maastricht 6202 AZ, The Netherlands; 7 Department of Internal Medicine, Seoul National University Hospital, 101, Daehak-ro Jongno-GU, Seoul 03080, South Korea; 8 Division of Thoracic Oncology, European Institute of Oncology, Via Ripamonti 435, Milan 20146, Italy; 9 Genentech Inc., DNA Way, South San Francisco, CA 94080, USA; 10 F. Hoffmann-La Roche Ltd, Grenzacherstrasse 124, Basel 4070, Switzerland; 11 Department of Medicine, University of California Irvine School of Medicine, 1001 Health Sciences Road, Irvine, CA 92617, USA

**Keywords:** alectinib, ALK positive, central nervous system, cumulative incidence rates, disease progression, non-small-cell lung cancer, phase II

## Abstract

**Background:**

We evaluated the cumulative incidence rate (CIR) of central nervous system (CNS) and non-CNS progression in alectinib-treated patients with anaplastic lymphoma kinase (*ALK*)-positive non-small-cell lung cancer (NSCLC) to determine the extent to which alectinib may treat or control CNS disease.

**Methods:**

Patients with crizotinib-pretreated locally advanced or metastatic disease received alectinib 600 mg orally twice daily in two phase II trials. All patients underwent baseline imaging and regular centrally reviewed scans.

**Results:**

At 24 months, the CIR for CNS progression was lower in patients without *vs* with baseline CNS metastases (8.0 *vs* 43.9%). Patients with baseline CNS disease and prior radiotherapy had a higher CIR of CNS progression than radiotherapy-naive patients (50.5 *vs* 27.4%) and a lower CIR of non-CNS progression (25.8 *vs* 42.5%). Adverse events leading to withdrawal occurred in 5.9% and 6.7% of patients with and without baseline CNS metastases, respectively.

**Conclusions:**

This analysis indicates a potential role for alectinib in controlling and preventing CNS metastases.

Rearrangements of the *anaplastic lymphoma kinase* (*ALK*) gene are indicative of a distinct subset of non-small-cell lung cancer (NSCLC), characterised by excellent responses to ALK-targeted therapy. Crizotinib was the first ALK inhibitor to receive US Food and Drug Administration (FDA) approval for the treatment of *ALK*-positive NSCLC (Food and Drug Administration press release, 2011).

Alectinib is a highly specific ALK inhibitor with good efficacy and tolerability demonstrated in phase II studies in patients with NSCLC harbouring *ALK* rearrangements (NP28673 [NCT01801111] and NP28761 [NCT01871805]) (Ou *et al*, 2016; Shaw *et al*, 2016). Based on these results, alectinib gained accelerated FDA approval for patients whose disease has progressed on, or who are intolerant to, crizotinib (Food and Drug Administration press release, 2015). Data from these and other studies suggest that alectinib is highly effective in treating central nervous system (CNS) metastases and in controlling the spread of the disease in the CNS (Seto *et al*, 2013; Gadgeel *et al*, 2016), a common site of progression with crizotinib (Gainor *et al*, 2013; Costa *et al*, 2015). Improving systemic and CNS efficacy, while maintaining good tolerability, is a clear goal in developing treatments for *ALK*-positive NSCLC.

This exploratory analysis evaluated cumulative incidence rates (CIRs) of CNS and non-CNS progression in alectinib-treated patients in the pivotal phase II studies (Ou *et al*, 2016; Shaw *et al*, 2016) to determine to what extent alectinib may treat and/or control the spread of the disease in the CNS in *ALK*-positive NSCLC.

## Materials and methods

### Study design

Methodology for both studies has been published (Ou *et al*, 2016; Shaw *et al*, 2016). Eligible patients were aged ⩾18 years with locally advanced or metastatic *ALK*-positive NSCLC, confirmed by an FDA-approved test, and an Eastern Cooperative Oncology Group performance status ⩽2. All patients had progressed on crizotinib. Patients with treated CNS metastases were eligible, provided any symptoms were stable for ⩾2 weeks before study entry; patients with untreated CNS metastases had to be asymptomatic. Patients received 600 mg oral alectinib twice daily until progression, death, or withdrawal.

### Assessments

Response was centrally evaluated using Response Evaluation Criteria in Solid Tumors v1.1. The study protocols stated that all patients, not only those with known CNS metastases, should undergo baseline brain imaging by magnetic resonance imaging, and have centrally reviewed CNS scans (every 8 weeks for NP28673; every 6 weeks for NP28761). Computed tomography of the chest and abdomen was also required.

The CNS progression events were analysed by cumulative incidence functions. The CNS progression was any new CNS lesion or progression of pre-existing CNS lesions *vs* baseline, according to an independent review committee (IRC). Non-CNS progression was any new lesion or progression of pre-existing lesions in areas outside the CNS, according to the same IRC. The CIRs for CNS progression, non-CNS progression, and death were calculated for patients with or without baseline CNS metastases using a competing risks method. The CIR analysis considers the first event in the competing risks setting. For example, if a patient had CNS progression before non-CNS progression or death, then the patient was considered as having a CNS progression event. Subgroup analysis of CIRs of CNS progression by prior radiation therapy status was undertaken in patients with baseline CNS metastases.

## Results

### Patients

Enrolment began in June 2013 for study NP28673 and in September 2013 for study NP28761; results are based on data cutoffs of 1 February 2016 and 22 January 2016, respectively. Baseline characteristics of the overall pooled population and the subgroups for this analysis were comparable and balanced between patients with or without baseline CNS metastases or prior radiation (Table 1).

### CIRs of progression

**Figure 1:**
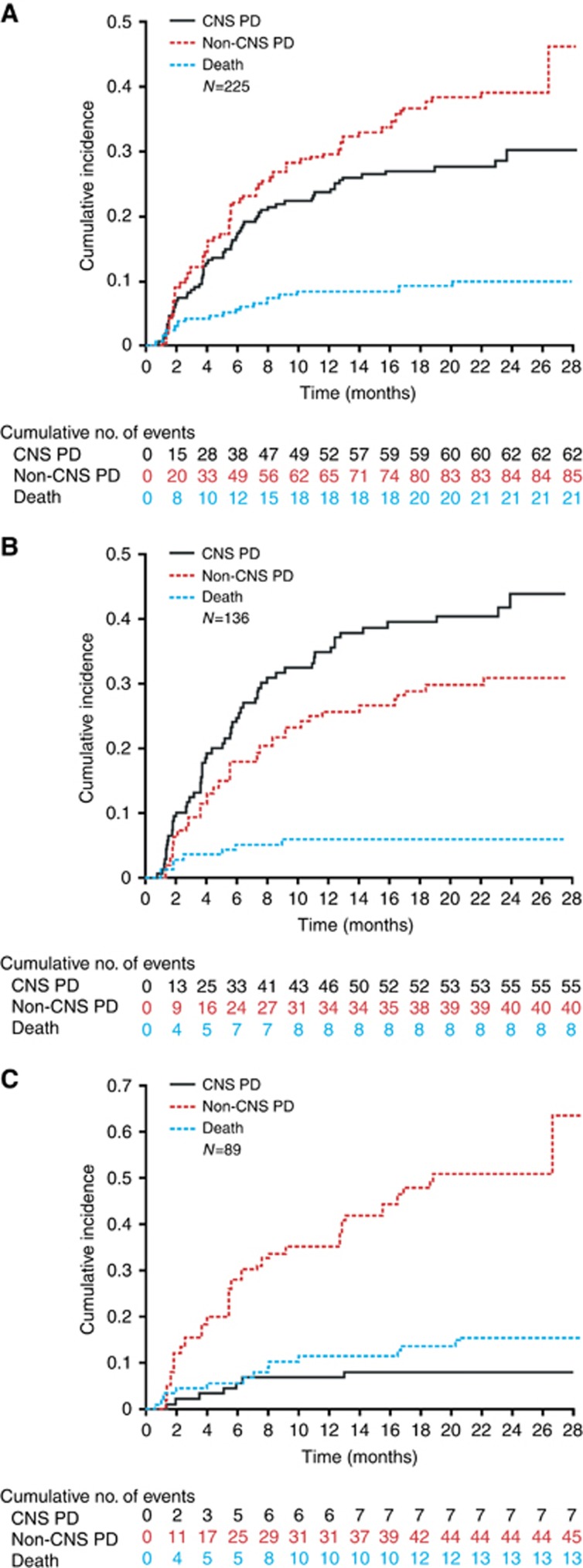
**Cumulative incidence rates for CNS progression, non-CNS progression, and death in alectinib-treated patients in the pivotal phase II studies.** (**A**) The overall pooled population, (**B**) patients with baseline CNS metastases, and (**C**) patients without baseline CNS metastases. CNS=central nervous system; PD=progressive disease.

For patients with baseline CNS metastases, CIRs of CNS and non-CNS progression at 24 months were 43.9% and 31.0%, respectively (Figure 1 and Table 2). In patients without baseline CNS metastases, the CIR of CNS progression was 8.0% at 24 months; these patients progressed at a higher rate in organs other than the CNS, as suggested by a higher CIR of non-CNS progression (50.9%).

Patients with baseline CNS metastases who had received prior radiotherapy had a higher CIR of CNS progression at 24 months than radiotherapy-naive patients (50.5% *vs* 27.4%, respectively) (Table 2). Data on the use of radiosurgery *vs* whole-brain radiotherapy were not collected.

### Safety

Median treatment duration was similar in the overall pooled population (43.57 weeks, range 2.4–89.0) and in patients with (44.79 weeks, range 3.0–85.7) or without (42.14 weeks, range 2.4–89.0) baseline CNS metastases.

Grade 3–5 adverse events (AEs) were reported in 45.6% of patients with baseline CNS metastases and in 32.6% of those without. Serious AEs occurred in 24.3% and 14.6% of patients, respectively, and AEs leading to treatment withdrawal occurred in 5.9% and 6.7% of patients, respectively.

## Discussion

The management of CNS metastases is essential for long-term outcome in *ALK*-positive NSCLC, with recent data identifying extended survival following radiotherapy and tyrosine kinase inhibitor treatment (Johung *et al*, 2016). The ALK inhibitor, alectinib, is highly active, both systemically (Ou *et al*, 2016; Shaw *et al*, 2016) and in the CNS (Seto *et al*, 2013; Gadgeel *et al*, 2016). Results of this exploratory analysis confirm these findings across all patient subgroups examined, and suggest a preventative effect of alectinib in the CNS.

These results must be considered in the context of the overall phase II study data. The curves of CIRs for CNS progression represent progression in one organ, whereas those for non-CNS progression represent progression at many possible sites. At 24 months, the CIR of CNS progression was only 43.9% in patients with baseline brain metastases, indicating that alectinib is active against CNS metastases. For patients without baseline CNS metastases, CIRs were higher for non-CNS progression than for CNS progression/death, suggesting that alectinib may prevent the spread of *ALK*-positive NSCLC to the brain, in addition to its activity against CNS metastases. If replicated in prospective analyses, these findings may provide an effective treatment strategy for patients with *ALK*-positive NSCLC to achieve long-term control of their systemic disease, by treating existing CNS lesions and protecting against new ones. These findings also support the hypothesised early administration of alectinib to maximise patient benefit. In patients with baseline CNS metastases, CIRs were higher for CNS progression than for non-CNS progression/death, reflecting the fact that these patients were at a higher risk of progressing in the brain, even if they received an active drug. It may also suggest a biologic specificity of *ALK*-rearranged tumours favouring the CNS as the elective site of progression.

Patients with baseline CNS metastases receiving radiotherapy had a higher CNS progression rate than untreated patients (data on the type of radiotherapy administered were not captured). This may be because patients who already received radiation carried the greatest burden or had symptomatic CNS disease requiring therapy.

This analysis is limited by its retrospective, exploratory nature, although data pooling enabled assessment in a broader patient population. The different CNS imaging schedules may have affected the CIRs, but the consistent trends over time suggest that any difference in rate between the study populations was minimal.

Our results confirm the potent ALK inhibition and robust efficacy of alectinib systemically and in the CNS. These findings may help to explain the longer progression-free survival observed with alectinib *vs* crizotinib in the global phase III ALEX study in patients with previously untreated *ALK*-positive NSCLC (Peters *et al*, 2017) and in the Japanese phase III J-ALEX study in the ALK inhibitor-naive setting (Hida *et al*, 2017). The ALEX study provided further evidence of the systemic and CNS efficacy of alectinib, with a complete CNS response rate of 38% in patients with measurable CNS lesions at baseline (Peters *et al*, 2017). In the intent-to-treat population, the CIR of CNS progression in ALEX, taking into account the competing risks of non-CNS progression and death, was 9.4 with alectinib *vs* 41.4% with crizotinib (Peters *et al*, 2017). When analysed according to baseline CNS metastases status, CIR trends observed for CNS PD *vs* non-CNS PD in ALEX were similar to those in the pooled analysis described above, reinforcing the CNS effectiveness of alectinib in preventing or delaying CNS metastases in *ALK*-positive NSCLC (Gadgeel *et al*, 2017). However, a more pronounced CNS prevention/delay effect was observed in ALK inhibitor-naive, front-line *ALK*-positive NSCLC patients as studied in ALEX; at 24 months, 16.0 and 4.6% of patients with or without CNS metastases at baseline, respectively, had CNS progression in ALEX, whereas 43.9 and 8% of patients had CNS PD in the phase II studies. Given the totality of available data, we propose a potential front-line role for alectinib in preventing/delaying the development of CNS metastases in *ALK*-positive NSCLC.

## Figures and Tables

**Table 1:** Baseline characteristics of the overall pooled population and the subgroups for this analysis by baseline metastases and prior radiation

**Characteristic**	**No baseline CNS metastases (*****n*****=89)**	**Baseline CNS metastases (*****n*****=136)**	**Baseline CNS metastases with prior CNS RT (*****n*****=95)**	**Baseline CNS metastases without prior CNS RT (*****n*****=41)**	**All patients (*****n*****=225)**
Median age, years (range)	54 (34–79)	51 (22–75)	50 (22–75)	54 (33–72)	53 (22–79)
Sex, *n* (%)					
Male	42 (47.2)	58 (42.6)	43 (45.3)	15 (36.6)	100 (44.4)
Female	47 (52.8)	78 (57.4)	52 (54.7)	26 (63.4)	125 (55.6)
ECOG PS, *n* (%)					
0	28 (31.5)	46 (33.8)	30 (31.6)	16 (39.0)	74 (32.9)
1	55 (61.8)	74 (54.4)	54 (56.8)	20 (48.8)	129 (57.3)
2	6 (6.7)	16 (11.8)	11 (11.6)	5 (12.2)	22 (9.8)
Histology, *n* (%)					
Adenocarcinoma	87 (97.8)	128 (94.1)	90 (94.7)	38 (92.7)	215 (95.6)
Other	2 (2.2)	8 (5.9)	5 (5.3)	3 (7.3)	10 (4.4)
Prior CTX, *n* (%)					
Yes	65 (73.0)	109 (80.1)	80 (84.2)	29 (70.7)	174 (77.3)
No	24 (27.0)	27 (19.9)	15 (15.8)	12 (29.3)	51 (22.7)

Abbreviations: CNS=central nervous system; CTX=chemotherapy; ECOG PS=Eastern Cooperative Oncology Group performance status; RT=radiotherapy.

**Table 2:** Cumulative incidence rates for CNS progression, non-CNS progression, and death^a^

Months	CIR, % (95% CI) CNS PD	CIR, % (95% CI) Non-CNS PD	CIR, % (95% CI) Death^a^
**All patients (*n*=225)**			
6	17.1 (12.2–22.1)	22.1 (16.6–27.6)	5.4 (2.4–8.3)
12	23.6 (18.0–29.2)	29.5 (23.5–35.5)	8.1 (4.5–11.8)
18	26.9 (21.0–32.8)	36.6 (30.2–43.0)	9.1 (5.3–12.9)
24	30.1 (23.3–36.8)	39.1 (32.5–45.7)	9.7 (5.8–13.7)
28	30.1 (23.3–36.8)	46.1 (33.3–58.9)	9.7 (5.8–13.7)
**Patients with no baseline CNS metastases (*n*=89) *vs* patients with baseline CNS metastases (*n*=136)**			
6	5.7 (0.8–10.5) *vs* 24.8 (17.4–32.1)	28.2 (18.8–37.6) *vs* 18.1 (11.5–24.6)	5.6 (0.8–10.4) *vs* 5.2 (1.5–9.0)
12	6.8 (1.5–12.0) *vs* 34.9 (26.7–43.0)	35.1 (25.1–45.0) *vs* 25.8 (18.3–33.3)	11.3 (4.7–17.9) *vs* 6.0 (2.0–10.0)
18	8.0 (2.3–13.6) *vs* 39.5 (31.2–47.9)	48.0 (37.5–58.5) *vs* 29.0 (21.2–36.7)	13.7 (6.5–20.9) *vs* 6.0 (2.0–10.0)
24	8.0 (2.3–13.6) *vs* 43.9 (34.7–53.1)	50.9 (40.2–61.5) *vs* 31.0 (23.0–39.1)	15.4 (7.6–23.2) *vs* 6.0 (2.0–10.0)
**Patients with baseline CNS metastases with prior CNS RT (*n*=95) *vs* patients with baseline CNS metastases without prior CNS RT (*n*=41)**			
6	30.3 (20.9–39.6) *vs* 12.2 (2.2–22.2)	16.2 (8.7–23.8) *vs* 22.2 (9.4–35.0)	5.4 (0.8–10.0) *vs* 4.9 (0.0–11.5)
12	41.4 (31.3–51.6) *vs* 19.8 (7.5–32.1)	19.6 (11.5–27.7) *vs* 39.9 (24.8–55.1)	5.4 (0.8–10.0) *vs* 7.4 (0.0–15.5)
18	44.8 (34.6–55.0) *vs* 27.4 (13.6–41.2)	23.0 (14.4–31.7) *vs* 42.5 (27.1–57.8)	5.4 (0.8–10.0) *vs* 7.4 (0.0–15.5)
24	50.5 (39.4–61.6) *vs* 27.4 (13.6–41.2)	25.8 (16.6–35.0) *vs* 42.5 (27.1–57.8)	5.4 (0.8–10.0) *vs* 7.4 (0.0–15.5)

Abbreviations: CI=confidence interval; CIR=cumulative incidence rate; CNS=central nervous system; PD=progressive disease; RT=radiotherapy.

a The CIR presents the first event in the competing risks setting, and thus patients who had first events of systemic PD or CNS PD would have death as a later event.

## References

[bib1] Costa DB, Shaw AT, Ou SH, Solomon BJ, Riely GJ, Ahn MJ, Zhou C, Shreeve SM, Selaru P, Polli A, Schnell P, Wilner KD, Wiltshire R, Camidge DR, Crinò L (2015) Clinical experience with crizotinib in patients with advanced ALK-rearranged non-small-cell lung cancer and brain metastases. J Clin Oncol 33: 1881–1888. 25624436 10.1200/JCO.2014.59.0539 PMC4451171

[bib2] Food and Drug Administration press release 26 August (2011) Available at: https://wayback.archive-it.org/7993/20170113081129/http://www.fda.gov/AboutFDA/CentersOffices/OfficeofMedicalProductsandTobacco/CDER/ucm270058.htm . Last accessed 17 January 2017.

[bib3] Food and Drug Administration press release 11 December (2015) Available at: http://www.fda.gov/NewsEvents/Newsroom/PressAnnouncements/ucm476926.htm . Last accessed 9 June 2017.

[bib4] Gadgeel SM, Peters S, Mok T, Shaw AT, Kim DW, Ou SI, Perol M, Dziadziuszko R, Ahn JS, Rosell R, Zeaiter A, Mitry E, Nueesch E, Balas B, Camidge DR (2017) Alectinib vs crizotinib in treatment-naïve ALK+ NSCLC: CNS efficacy results from the ALEX study. ESMO Abstract 1298O. Ann Oncol 28(suppl_5): v605–v649.

[bib5] Gadgeel SM, Shaw AT, Govindan R, Gandhi L, Socinski MA, Camidge DR, De Petris L, Kim DW, Chiappori A, Moro-Sibilot DL, Duruisseaux M, Crino L, De Pas T, Dansin E, Tessmer A, Yang JC, Han JY, Bordogna W, Golding S, Zeaiter A, Ou SI (2016) Pooled analysis of CNS response to alectinib in two studies of pretreated patients with *ALK*-positive non-small-cell lung cancer. J Clin Oncol 34: 4079–4085. 27863201 10.1200/JCO.2016.68.4639 PMC7845943

[bib6] Gainor JF, Ou SH, Logan J, Borges LF, Shaw AT (2013) The central nervous system as a sanctuary site in ALK-positive non-small-cell lung cancer. J Thorac Oncol 8: 1570–1573. 24389440 10.1097/JTO.0000000000000029

[bib7] Hida T, Nokihara H, Kondo M, Azuma K, Seto T, Takiguchi Y, Nishio M, Yoshioka H, Imamura F, Hotta K, Watanabe S, Goto K, Satouchi M, Kozuki T, Shukuya T, Nakagawa K, Mitsudomi T, Yamamoto N, Asakawa T, Asabe R, Tanaka T, Tamura T (2017) Alectinib versus crizotinib in patients with *ALK*-positive non-small-cell lung cancer (J-ALEX): an open-label, randomised phase 3 trial. Lancet 390: 29–39. 28501140 10.1016/S0140-6736(17)30565-2

[bib8] Johung KL, Yeh N, Desai NB, Williams TM, Lautenschlaeger T, Arvold ND, Ning MS, Attia A, Lovly CM, Goldberg S, Beal K, Yu JB, Kavanagh BD, Chiang VL, Camidge DR, Contessa JN (2016) Extended survival and prognostic factors for patients with ALK-rearranged non-small-cell lung cancer and brain metastasis. J Clin Oncol 34: 123–129. 26438117 10.1200/JCO.2015.62.0138 PMC5070549

[bib9] Ou SH, Ahn JS, De Petris L, Govindan R, Yang JC, Hughes B, Lena H, Moro-Sibilot D, Bearz A, Ramirez SV, Mekhail T, Spira A, Bordogna W, Balas B, Morcos PN, Monnet A, Zeaiter A, Kim DW (2016) Alectinib in crizotinib-refractory ALK-rearranged non-small-cell lung cancer: a phase II global study. J Clin Oncol 34: 661–668. 26598747 10.1200/jco.2015.63.9443

[bib10] Peters S, Camidge DR, Shaw AT, Gadgeel S, Ahn JS, Kim DW, Ou SI, Perol M, Dziadziuszko R, Rosell R, Zeaiter A, Mitry E, Golding S, Balas B, Noe J, Morcos PN, Mok T ALEX Trial Investigators (2017) Alectinib versus crizotinib in untreated *ALK*-positive non-small-cell lung cancer. N Engl J Med 377: 829–838. 28586279 10.1056/NEJMoa1704795

[bib11] Seto T, Kiura K, Nishio M, Nakagawa K, Maemondo M, Inoue A, Hida T, Yamamoto N, Yoshioka H, Harada M, Ohe Y, Nogami N, Takeuchi K, Shimada T, Tanaka T, Tamura T (2013) CH5424802 (RO5424802) for patients with ALK-rearranged advanced non-small-cell lung cancer (AF-001JP study): a single-arm, open-label, phase 1-2 study. Lancet Oncol 14: 590–598. 23639470 10.1016/S1470-2045(13)70142-6

[bib12] Shaw AT, Gandhi L, Gadgeel S, Riely GJ, Cetnar J, West H, Camidge DR, Socinski MA, Chiappori A, Mekhail T, Chao BH, Borghaei H, Gold KA, Zeaiter A, Bordogna W, Balas B, Puig O, Henschel V, Ou SH study investigators (2016) Alectinib in ALK-positive, crizotinib-resistant, non-small-cell lung cancer: a single-group, multicentre, phase 2 trial. Lancet Oncol 17: 234–242. 26708155 10.1016/S1470-2045(15)00488-X PMC4752892

